# Prognostic role of magnetic resonance imaging of the abdomen with intravenous contrast and magnetic resonance cholangiopancreatography in primary sclerosing cholangitis

**DOI:** 10.1590/0100-3984.2023.0041

**Published:** 2023

**Authors:** Roy López Grove, Florência Vespa, Martina Aineseder, Alejandra Villamil, Juan Carlos Spina

**Affiliations:** 1 Hospital Italiano de Buenos Aires, Ciudad Autónoma de Buenos Aires, Argentina.

**Keywords:** Cholangitis, Cholangiography, Magnetic resonance imaging, Cholangiopancreatography, magnetic resonance, Colangiografia, Ataxia cerebelar, Ressonância magnética, Colangiopancreatografia por ressonância magnética

## Abstract

**Objective:**

To evaluate the usefulness of Anali scores, determined by magnetic resonance imaging, for predicting the prognosis of primary sclerosing cholangitis (PSC) and to analyze interobserver variability, as well as to assess the impact of periportal edema and heterogeneous signal intensity on diffusion-weighted imaging of the liver.

**Materials and Methods:**

This was a retrospective cohort study of 29 patients with PSC and baseline magnetic resonance imaging. Anali scores, without gadolinium (0-5 points) and with gadolinium (0-2 points), were calculated by two radiologists. Clinical end-points included liver transplantation, cirrhotic decompensation, and death. We calculated intraclass correlation coefficients (ICCs) for interobserver agreement on the Anali scores, performed Kaplan-Meier survival analysis comparing event-free survival among the score strata, and calculated the areas under receiver operating characteristic curves to determine sensitivity and specificity.

**Results:**

Among the patients with a clinical event, the median Anali score was 4 (interquartile range [IQR], 2-5) without gadolinium and 2 (IQR, 1–2) with gadolinium, compared with 1 (IQR, 1.0–2.5) and 1 (IQR, 0.25–1.0), respectively, among those without a clinical event. The ICC was 0.79 (95% confidence interval: 0.57–0.91) for the Anali score with gadolinium and 0.99 (95% confidence interval: 0.98–0.99) for the Anali score without gadolinium. Periportal edema and heterogeneous signal intensity in the liver on diffusion-weighted imaging showed no statistical impact on clinical events (*p* = 0.65 and *p* = 0.5, respectively).

**Conclusion:**

Anali scores correlate with clinical events in PSC, with a high level of interobserver agreement.

## Abstract (pt)

**Objetivo:**

Avaliar a utilidade dos escores Anali determinados por ressonância magnética para prever o prognóstico da colangite esclerosante primária (CEP), analisar a variabilidade interobservador e avaliar o impacto do edema periportal e do sinal heterogêneo do fígado em imagens ponderadas por difusão.

**Materiais e Métodos:**

Estudo retrospectivo de coorte de 29 pacientes com CEP e ressonância magnética de base. Os escores Anali sem gadolínio (0 a 5 pontos) e com gadolínio (0 a 2 pontos) foram calculados por dois radiologistas. Os desfechos clínicos incluíram transplante de fígado, descompensação cirrótica ou morte. Foram realizados coeficiente de correlação intraclasse (CCI) para a concordância interobservador com relação ao escore Anali, análise de sobrevivência de Kaplan-Meier comparando o tempo livre de eventos de acordo com o escore, e área sob a curva característica de operação do receptor para sensibilidade e especificidade.

**Resultados:**

Nos pacientes com evento clínico, a mediana do escore Anali sem gadolínio foi 4 (intervalo interquartil [IIQ]: 2–5) e com gadolínio foi 2 (IIQ: 1–2), enquanto nos pacientes sem evento clínico o escore sem gadolínio foi 1 (IIQ:1–2,5) e com gadolínio foi 1 (IIQ: 0,25–1). A concordância interobservador com gadolínio foi CCI = 0,79 (intervalo de confiança 95%: 0,57–0,91) e sem gadolínio foi CCI = 0,99 (intervalo de confiança 95%: 0,98-0,99). O edema periportal (p = 0,65) e o sinal heterogêneo do fígado nas imagens ponderadas por difusão (p = 0,5) não apresentaram impacto nos eventos clínicos.

**Conclusão:**

Os escores Anali se correlacionam com eventos clínicos na CEP, com alto grau de concordância interobservador.

## INTRODUCTION

Primary sclerosing cholangitis (PSC) is a chronic cholestatic liver disease of unknown etiology, characterized by inflammation and obliterative fibrosis of the biliary tree. Although the course is highly variable, PSC is often progressive, leading to biliary cirrhosis and its complications. Currently, there is no effective medical therapy and liver transplantation is the only therapeutic intervention that prolongs the life of patients with end-stage liver disease^(1)^.

In patients with otherwise unexplained biochemical cholestasis, a diagnosis of PSC is made when magnetic resonance cholangiopancreatography (MRCP) shows multifocal stenosis and segmental dilatations, assuming that causes of secondary sclerosing cholangitis (especially immunoglobulin G-associated cholangitis) have been excluded^(2,3)^.

Although endoscopic retrograde cholangiopancreatography was previously the diagnostic procedure of choice, it has been supplanted by MRCP because the latter has a higher diagnostic yield, is more cost-effective, and is non-invasive^(1,4)^. Despite the fact that imaging is central to the diagnosis of PSC, its potential for identifying prognostic markers has been little explored.

The most widely used prognostic model in PSC is the Mayo risk score, which is based on patient age, bilirubin, aspartate aminotransferase, variceal bleeding, and albumin^(5)^. Recent studies have attempted to detect an association between MRI findings with biochemical scores and MR elastography to assess its usefulness in patients with PSC^(6,7)^.

Ruiz et al.^(2)^ described the process of predicting radiological progression in patients with PSC. The authors predicted such progression by determining the presence, at baseline, of severe intrahepatic bile duct dilatation, liver dysmorphism, portal hypertension, and heterogeneity of parenchymal enhancement after injection of a gadoliniumbased contrast agent. On the basis of the combination of those features, two MRI progression risk scores, known as the Anali without and with gadolinium scores, were developed with the aim of predicting radiological progression in such patients^(1)^. Some studies have evaluated those scores and have obtained promising results^(1,8)^. However, to our knowledge, there have been no studies using MRI to analyze the prognosis of patients with PSC in South America or evaluating other possible signs of inflammation such as periportal edema on unenhanced images and heterogeneous signal intensity on diffusion-weighted imaging (DWI) of the liver parenchyma, neither of which were taken into consideration when the Anali scores were created^(9,10)^.

The objective of the present study was to evaluate the usefulness of the Anali score for MRI of the abdomen with intravenous contrast and MRCP to predict the prognosis of patients with PSC, to evaluate the usefulness of periportal edema and heterogeneous signal intensity in the liver parenchyma on DWI sequences as potential signs of liver inflammation, and to analyze the interobserver variability for the assessment of this score.

## MATERIALS AND METHODS

### Study population

This was a retrospective cohort study in which we analyzed the cases of all patients diagnosed with PSC and followed at our institution between 2009 and 2020. The study was approved by the research ethics committee of our institution. Because of the retrospective nature of the study, the requirement for informed consent was waived.

The inclusion criteria were as follows: being > 18 years of age at the time of MRI/MRCP; having been diagnosed with large-duct PSC; and relevant MRI/MRCP images being available for review. The diagnosis of large-duct PSC was based on the clinical presentation of cholestasis, MRCP images showing multifocal stenosis and segmental dilations, and the exclusion of causes of secondary sclerosing cholangitis^(11)^. The MRI/MRCP study acquired closest to the diagnosis of PSC was considered the baseline study, and the date of that study was considered the inclusion date.

Patients for whom the MRI/MRCP images were of poor quality because of artifacts were excluded, as were those with overlap syndrome between autoimmune hepatitis and PSC; those with a history of liver transplantation, bile duct surgery or liver comorbidities (viral hepatitis, alcoholic liver disease, or nonalcoholic steatohepatitis); and those with secondary sclerosing cholangitis, cholangiocarcinoma, hepatocellular carcinoma, or decompensated cirrhosis at the time of inclusion.

### MRI technique

Fasting for 4–6 h before MRI was indicated. All MRI studies were performed in 1.5-T scanners (Magnetom Avanto or Essenza; Siemens Healthineers, Erlangen, Germany) with phased-array coils. The imaging protocol was as follows: axial T2-weighted sequences, with and without fat saturation; a coronal T2-weighted sequence; coronal T1-weighted in-phase and out-of-phase DWI sequences (with b-values of 50, 400, and 800 s/mm^2^); contrast-enhanced axial and coronal Tl-weighted volume interpolated breath-hold examination (VIBE) sequences with fat saturation; and two-dimensional (2D) and three-dimensional (3D) fast spin-echo pulse MRCP sequences. For the contrast-enhanced sequences, a gadolinium-based contrast agent (meglumine gadoterate) was administered intravenously at a standard dose of 0.1 mmol/kg body weight.

### Imaging analysis

All MRI scans were reviewed by two abdominal radiologists with 3 and 15 years of experience, respectively, working independently, who were blinded to all patient data except for the diagnosis of PSC. The two Anali scores, without and with gadolinium, were calculated according to previous reports, as follows: the Anali without gadolinium score was calculated as 1 × intrahepatic bile duct dilatation + 2 × liver dysmorphism + 1 × portal hypertension, with a possible score range of 0-5 points; and the Anali with gadolinium score was calculated as 1 × liver dysmorphism + 1 × heterogeneous enhancement of the liver parenchyma in the arterial phase, with a possible score range of 0–2 points.

Intrahepatic bile duct dilatation was scored according to the measurement of the duct at its maximum diameter, as follows: 0 points if it was < 3 mm; 1 point if it was 3–5 mm; and 2 points if it was > 5 mm. The most dilated segment was chosen for measurement on 3D MRCP.

Portal hypertension, liver dysmorphism, and heterogeneous enhancement of the liver parenchyma in the arterial phase were each scored as 0 points if absent and 1 point if present. Portal hypertension was determined by the presence of portosystemic shunts, splenomegaly, or ascites. Liver dysmorphism was defined by the presence of significant atrophy of the right or left hepatic lobe, an above-normal modified caudate/right lobe ratio^(12)^, and a markedly lobular liver surface.

Abdominal MRI findings were also evaluated for the presence of periportal edema and heterogeneous signal intensity in the liver parenchyma, to find new MRI signs to assess inflammatory liver disease activity without gadolinium, which could increase the sensitivity or specificity of the Anali score^(2)^. Periportal edema was defined as periportal halos around the central portal veins or their peripheral branches showing increased periportal signal intensity on a T2-weighted sequence and periportal enhancement after intravenous gadolinium administration. That finding has been reported in 40–68% of cases^(13,14)^. The presence of heterogeneous signal intensity in the liver parenchyma was evaluated on DWI^(15,16)^.

### Clinical data collection

Clinical data were collected from patient records and were anonymized. The following data were collected: age at diagnosis of PSC; association with any inflammatory bowel disease; the biochemical analysis closest to the date of inclusion and to that of the first MRI at the institution (up to three months before or after), including total bilirubin, alkaline phosphatase, aspartate aminotransferase, and alanine aminotransferase; and clinical events occurring after inclusion, such as liver transplantation, decompensation of cirrhosis (as evidenced by hemorrhagic varices), ascites, hepatic encephalopathy, hepatorenal syndrome, and death.

### Statistical analysis

The level of interobserver agreement for the Anali scores was evaluated by calculating the intraclass correlation coefficients (ICCs) for the scores without and with gadolinium. That level, based on the ICC, was stratified as follows^(17)^: 0.01–0.20, no or slight agreement; 0.21–0.40, fair agreement; 0.41–0.60, moderate agreement; 0.61–0.80, substantial agreement; and 0.81–1.00, almost perfect agreement. We also calculated the kappa statistic to determine the level of interobserver agreement for the individual signs. In addition, we performed Kaplan-Meier survival analysis comparing event-free survival according to the Anali scores. Furthermore, receiver operating characteristic (ROC) curves were generated and the areas under the curve (AUCs) were calculated to determine the sensitivity and specificity of the Anali scores without and with gadolinium.

The Shapiro-Wilk test was used in order to determine the normality of the data, with a result of *p* = 0.02, which is consistent with a nonparametric distribution. Patient characteristics and biochemical variables were summarized as medians and interquartile ranges (IQRs) or as absolute numerical values and percentages. Continuous and categorical variables were compared between subjects by using the appropriate statistical tests according to the data distribution. The Mann-Whitney U test was used in order to compare numerical variables. The level of statistical significance was set at *p* < 0.05.

## RESULTS

We evaluated the cases of 29 patients with a diagnosis of large-duct PSC. The clinical characteristics of the patients are summarized in Table 1. Of the 29 patients evaluated, 12 (41.4%) experienced a clinical event during follow-up: liver transplantation, in seven; and decompensation of cirrhosis, in five. The median time between the MRI and the clinical event was 30.5 months (mean, 41 ± 23 months). Comparing the groups of patients with and without a clinical event, we found that the median serum total bilirubin concentration was significantly higher in the former *(p* = 0.003), whereas the median serum albumin concentration was significantly higher in the latter (*p* = 0.008). Although the median age was higher in the group of patients with a clinical event, the difference was not statistically significant (*p* = 0.14). Only four patients were the same age at baseline MRI/MRCP as at the diagnosis of PSC.

**Table 1:** Main clinical characteristics of the patients.

	Clinical event		
Characteristic	No (n = 17)	Yes (n = 12)	P
Male, n (%)	10 (59)	5 (42)	0.4
Age at diagnosis (years), median (IQR)	27 (12–76)	44 (6–70)	0.14
Age at MRI (years), median (IQR)	35 (18–82)	58 (18–70)	0.46
Underlying disease, n (%)			
Ulcerative colitis	15 (88)	9 (75)	0.62
Celiac disease	2 (12)	0 (0)	–
Total bilirubin (mg/dL), median (IQR)	0.7 (0.3–3.1)	1.7 (0.5-23.0)	0.003
Alkaline phosphatase (IU/L), median (IQR)	229.0 (72.0–681.0)	277.5 (108.0–707.0)	0.9
Aspartate aminotransferase (IU/L), median (IQR)	42 (16–286)	86 (21–212)	0.06
Alanine aminotransferase (IU/L), median (IQR)	91.0 (9.0–283.0)	81.5 (22.0–237.0)	0.7
Albumin (g/dL), median (IQR)	4.0 (3.2–4.8)	3.4 (3.0–3.8)	0.008

**Figure 1:**
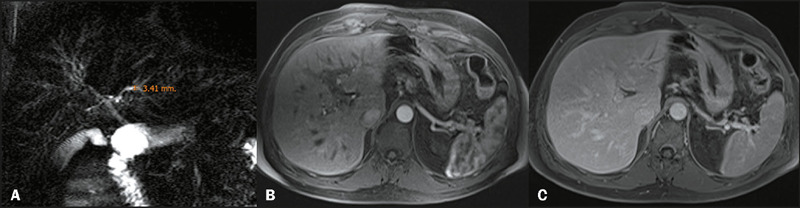
A 48-year-old man with a known history of ulcerative colitis and PSC. Coronal projection of a 2D MRCP scan **(A),** together with axial contrast-enhanced fat-suppressed T1-weighted sequences in the arterial and portal venous phases **(B** and **C,** respectively), showing a maximum ductal diameter between 3 mm and 5 mm, with normal liver morphology, arterial enhancement, and no signs of portal hypertension. The Anali scores without and with gadolinium were calculated as 1 and 0, respectively, by both observers. This patient did not experience a clinical event.

**Figure 2:**
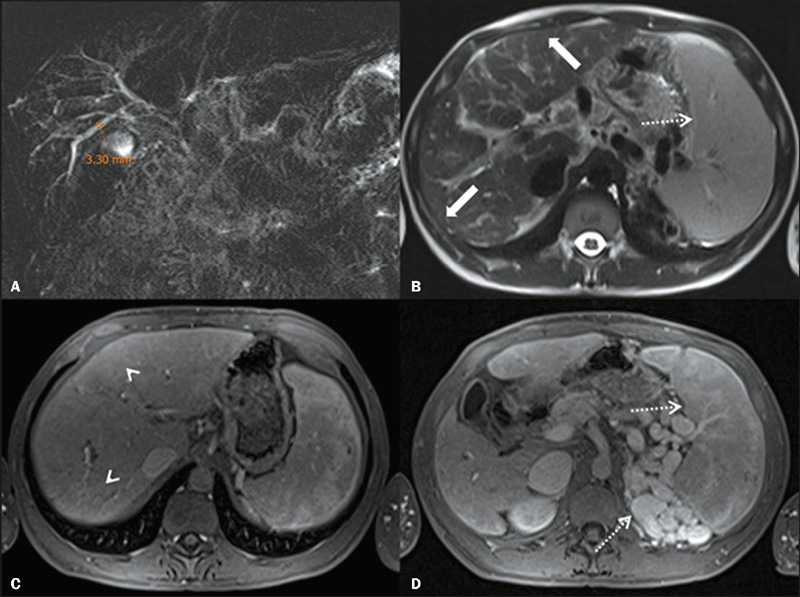
Coronal projection of a 2D MRCP scan **(A)** and an axial T2-weighted sequence **(B),** together with contrast-enhanced fat-suppressed T1-weighted sequences in the arterial and portal venous phases **(C** and **D,** respectively), of a 33-year-old female patient with PSC. The Anali scores without and with gadolinium were calculated as 4 and 2, respectively, by both observers. Note the liver dysmorphism (arrows), maximum ductal diameter of 3-5 mm, heterogeneity of arterial enhancement of the liver parenchyma (arrowheads), and signs of portal hypertension (perisplenic circulation and splenomegaly, dotted arrows).

Unenhanced and contrast-enhanced abdominal MRI and MRCP were performed in all 29 patients (Figures 1 and 2). The ICCs for the Anali scores without and with gadolinium were 0.99 (95% confidence interval [CI]: 0.98–0.99) and 0.79 (95% CI: 0.57–0.91), respectively (*p* < 0.0001 for both). Among the patients with a clinical event, the median Anali scores without and with gadolinium were 4 and 2, respectively, compared with 1 and 1, respectively, among those with no clinical event (*p* = 0.029 and *p* = 0.012, respectively).

**Figure 3:**
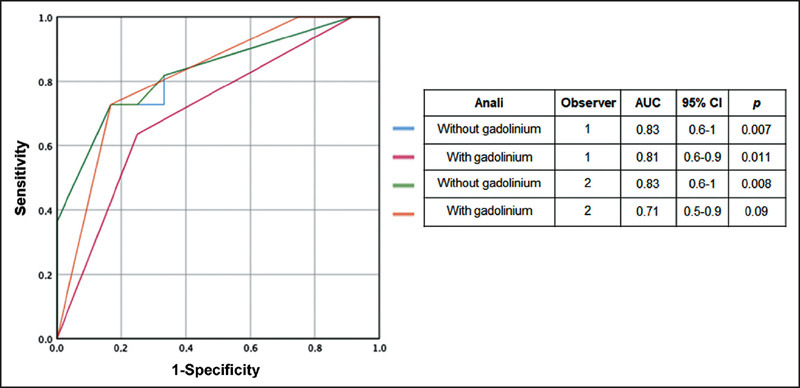
The ROC curves, with AUCs, for the sensitivity and specificity of the Anali scores with and without gadolinium, as determined by each observer.

The optimal cutoff for the Anali score without gadolinium was found to be 1.5, with a sensitivity of 0.81 and specificity of 0.67 for both observers, whereas that for the Anali score with gadolinium was also found to be 1.5, although it had a sensitivity of 0.72 and specificity of 0.83 for the first observer and a sensitivity of 0.63 and specificity of 0.75 for the second observer. The ROC curves for the Anali scores with and without gadolinium are shown in Figure 3. The mean AUC of the Anali score without gadolinium for predicting radiologic progression was 80.2 ± 4.0% for observer 1 and 83.0 ± 17.0% for observer 2, whereas the AUC of the Anali score with gadolinium was 70.8 ± 21.7% for observer 1 and 81.4% ± 17.9% for observer 2.

**Figure 4:**
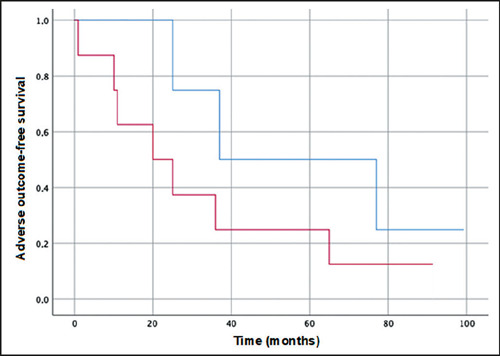
Kaplan-Meier curves for event-free survival, by Anali score. The blue line represents patients with low Anali scores (< 2 without gadolinium and ≤ 1 with gadolinium), and the red line represents patients with high Anali scores (> 2 without gadolinium and > 1 with gadolinium). An event was defined as liver transplantation, decompensation of cirrhosis, or liver disease-related death.

In the Kaplan-Meier survival analysis (Figure 4), event-free survival was found to be longer among the patients with lower Anali scores (0–2) than among those with higher Anali scores (3–5)—59 months (95% CI: 25–93) versus 32 months (95% CI: 10–53).

The kappa statistic for interobserver agreement was 0.016 (95% CI: 0–0.7; *p* = 0.52) for dilatation of the intrahepatic bile ducts, 0.92 (95% CI: 0.76-1.00; *p* < 0.00001) for portal hypertension, 1.00 (95% CI: 1.00-1.00; *p* < 0.00001) for liver dysmorphism, and 0.25 (95% CI: 0-0.76; *p* = 0.2) for heterogeneous enhancement of the liver parenchyma in the arterial phase. The main interobserver discrepancy was for dilation of the biliary tract, followed by heterogeneous enhancement of the liver parenchyma. There was discordance between the observers for portal hypertension in only one case, whereas there was no discordance in the analysis of liver dysmorphism in any of the cases.

Increased periportal signal intensity on the T2-weighted sequence was seen in only three patients, two of whom had a clinical event (*p* = 0.65), and the presence of heterogeneous signal intensity in the liver parenchyma on DWI was present in 25 patients, 11 of whom had a clinical event (*p* = 0.5).

A high frequency of ulcerative colitis was observed in both groups: in 15 of the 17 patients with a clinical event; and in 9 of the 12 without a clinical event. The occurrence of a clinical event was associated with the presence of associated inflammatory bowel disease (Fisher’s exact test, *p* = 0.33).

## DISCUSSION

Our study showed a high level of interobserver agreement for the Anali scores without and with gadolinium. Those scores were found to be predictive of clinical events, which were also associated with higher age and higher bilirubin levels.

The main interobserver discrepancy was for the parameter of intrahepatic bile duct dilatation, for which there was discordance in three cases in our sample, probably because it is a quantitative variable with strict limits on scoring values. A small difference in the measurement of bile duct diameter can change the category, especially in the measurement between 3 mm and 5 mm. The heterogeneity of parenchymal enhancement also presented discordance, because its assessment depends on the perception of the observer. There is no objective or quantitative analysis for this parameter, the analysis being especially difficult when the heterogeneity is milder and being dependent on the level of experience of the observer. Heterogeneous hepatic hyperenhancement in the arterial phase is associated with acute liver inflammation and therefore with the development of fibrosis, which is known to be reversible.

The assessment of liver dysmorphism is also subjective, because subtle lobulations of the margins that are interpreted as normal by one observer could be interpreted as pathological by another. There is no scale to objectively evaluate this parameter, and its misinterpretation can lead to a change in the Anali score. However, in our sample, there was no significant discrepancy in the analysis of this variable, possibly due to the fact that there were marked lobulations in the affected patients.

In the present study, there were no differences between the two observers for the signs of portal hypertension on the contrast-enhanced studies. However, it is more difficult to evaluate esophageal or para-esophageal varices and patency of the paraumbilical vein on unenhanced studies. It can also be more difficult for a less experienced observer to assess perisplenic collateral circulation or collaterals in other regions.

In a recent study conducted by Grigoriadis et al.^(8)^, Anali scores with and without gadolinium showed poor to moderate interobserver agreement, the main differences of opinion being in the assessment of liver dysmorphism and heterogeneous enhancement of the liver parenchyma. Our findings are in agreement with theirs in terms of the latter parameter, the analysis of which is subjective and is more dependent on the observer level of experience. Although there have been studies evaluating the interob-server agreement on MRI/MRCP scans in patients with PSC, which also showed poor interobserver agreement, those studies did not specifically evaluate the parameters used here^(18,19)^. This discrepancy between studies could be explained by differences in the characteristics of the populations studied. Like Grigoriadis et al.^(8)^, we showed that both Anali scores were significantly associated with the occurrence of clinical events.

Cazzagon et al.^(20)^ attempted to demonstrate the usefulness of MRI risk scores and liver stiffness in predicting clinical outcomes in PSC. Their study sample included 162 patients, 40 of whom experienced a clinical event. The authors identified a significant correlation between liver stiffness and a high Anali score without gadolinium, both of which were independently associated with the occurrence of an adverse outcome. The combined use of those two thresholds allowed the authors to stratify the patients by the risk of adverse outcomes (low, medium, or high). In the present study, we did not analyze liver stiffness, because there were no available data regarding that parameter at the time of MRI in most patients.

In our study sample, high periportal signal intensity on T2-weighted sequences and heterogeneous signal intensity in the liver parenchyma on DWI did not differ significantly between the patients with and without a clinical event or among the Anali score strata. Only a few of the patients (n = 3) showed increased periportal signal intensity on T2-weighted sequences. The absence of periportal abnormalities does not exclude periportal inflammation^(21)^. We also found that DWI was not useful in the assessment of fibrotic involvement of the liver parenchyma, which is in agreement with the findings of other authors^(22,23)^.

Early peribiliary hyperenhancement on multiphasic contrast-enhanced MRI of the liver and biliary tree has been significantly associated with higher Mayo risk scores and may suggest a poorer outcome and decreased survival in patients with PSC^(24)^. Early peribiliary hyperenhancement could also be attributed to ongoing cholangitis, given that heterogeneous hepatic hyperenhancement in the arterial phase has been correlated with acute liver inflammation and therefore with a worse prognosis. The Anali score does not consider this factor; its analysis and consideration as a marker of acute inflammation could be useful, as could that of heterogeneous hepatic hyperenhancement in the arterial phase, unlike the rest of the findings, which are indicative of chronic disease.

Although our data are encouraging, it should be noted that our study has some limitations. The retrospective design could have introduced a selection bias, which could explain why there was a significant difference between age at onset and age at first MRI in some of the patients. In addition, the sample size was small and there were few clinical events during follow-up. A larger patient sample and a longer observation period could influence that parameter.

## CONCLUSION

Anali scores determined on MRI showed a correlation with clinical outcomes in patients with PSC, and there was a high level of interobserver agreement in our study sample. Although further studies with larger numbers of patients and follow-up are needed, these scores proved to be useful as prognostic factors in such patients.
